# Whole‐Genome Resequencing Provides Novel Insights Into the Genetic Diversity, Population Structure, and Patterns of Runs of Homozygosity in Mud Crab (*Scylla paramamosain*)

**DOI:** 10.1111/eva.70153

**Published:** 2025-09-03

**Authors:** Xiyi Zhou, Min Ouyang, Yin Zhang, Mhd Ikhwanuddin, Hongyu Ma, Shaopan Ye

**Affiliations:** ^1^ Guangdong Provincial Key Laboratory of Marine Biotechnology Shantou University Shantou China; ^2^ International Joint Research Center for the Development and Utilization of Important Mariculture Varieties Surrounding the South China Sea Region Shantou University Shantou China; ^3^ STU‐UMT Joint Shellfish Research Laboratory Shantou University Shantou China; ^4^ Higher Institute Centre of Excellence (HICoE), Institute of Tropical Aquaculture and Fisheries Universiti Malaysia Terengganu Kuala Nerus Terengganu Malaysia

**Keywords:** genetic diversity, runs of homozygosity, *S. paramamosain*, whole genome resequencing

## Abstract

Mud crab (*Scylla paramamosain*) is an economically important aquaculture crustacean species in China and Southeast Asia countries. However, the catches of wild mud crabs declined sharply due to overfishing and environmental pollution. Therefore, it is necessary to understand the current genetic resources and population history of mud crab (*S. paramamosain*), which would provide appropriate guidelines for genetic resource management and breeding programs. To achieve this goal, a total of 146 mud crabs from four geographic populations in the southeast coast of China were collected for whole genome resequencing to investigate the genetic diversity, population genetic structure, and runs of homozygosity (ROHs). Results showed that the nucleotide diversity (π) ranged from 0.00157 to 0.00160, with observed heterozygosity (0.248–0.257) approximately equal to expected heterozygosity (0.260–0.265), indicating that these populations were near Hardy–Weinberg equilibrium, albeit with relatively low polymorphism. The results of PCA, population structure, phylogenetic tree, and linkage disequilibrium (LD) analysis consistently indicated weak genetic differentiation among different geographic populations. ROHs detection revealed 47,142 ROHs in mud crabs, with over 60% shorter than 0.1 Mb. Moreover, the average genomic inbreeding coefficient estimated by ROHs (*F*_ROH_ = 0.0293) and homozygous sites (*F*_HOM_ = 0.0389) suggested relatively low inbreeding in mud crab populations. Notably, 29 candidate genes were identified in potential ROH islands, including growth and development‐related genes (*IARS* and *UNC79*), which may play an important role in the adaptive evolution of mud crabs. Overall, our results would provide valuable insights for conserving, managing, and improving the genetic resources of mud crabs (*S. paramamosain*).

## 1 Introduction

Genetic diversity plays a crucial role in species adaptation to environmental changes, with significant implications for various biological applications including selective breeding programs, conservation strategies, and resource management (Fageria and Rajora 2013). High genetic diversity supports long‐term sustainability by maintaining adaptive potential, reducing the risk of inbreeding depression, and enabling responses to challenges such as disease outbreaks and environmental changes (Gibson 2022; Teixeira and Huber 2021). Therefore, genetic diversity analysis is indispensable for understanding the evolutionary processes of species and provides essential insights that inform biodiversity conservation and the sustainable management of natural resources. Nowadays, genetic diversity analysis has been widely used in aquaculture species for the preservation and utilization of germplasm resources.

The mud crab (*Scylla paramamosain*) is one of the most economically important aquaculture crustacean species, widely distributed along the southeastern coastal region of China, and renowned for high nutritional value and fast growth rate (Cui, Fang, et al. 2021; Esmaeili et al. 2024; Zhang et al. 2025). In China, the mud crab has been farmed for over 100 years, with the earliest artificial farming dating back to the 1980s (Liew et al. 2023; Shen and Lai 1994). According to the China Fisheries Statistical Yearbook (Bureau of Fisheries et al. 2024), the yield of artificial breeding of mud crab in China has reached 157.012 thousand tons in 2023, with a breeding scale of 24.809 thousand hectares. However, mud crab farming still mainly relies on wild‐caught seed crabs (Apine et al. 2023; Ye et al. 2025). Due to overfishing and environmental pollution (Waqas et al. 2024), the national marine capture production of mud crab decreased from 69.025 thousand tons in 2022 to 67.079 thousand tons in 2023 (Bureau of Fisheries et al. 2024), which may decrease the diversity of germplasm resources and the capture production of seed crabs of mud crab, thus seriously restricting the sustainable development of mud crab farming (Wang et al. 2020). Therefore, it is necessary to perform genetic diversity analysis to understand the genetic resources of mud crabs over time, which would provide appropriate guidelines for resource management and breeding programs.

Hitherto, the genetic diversity analysis of mud crabs in China has been performed based on various molecular markers, including inter‐simple sequence repeat (ISSR), mitochondrial DNA, and microsatellite markers. For instance, a high level of genetic diversity and a low level of differentiation were indicated in mud crabs from six geographic populations on Hainan Island of China based on the mtDNA COI gene sequence (Ma et al. 2011). Besides, similar results were also found in mud crabs along the southeastern coast of China using nine microsatellite markers (Ma et al. 2012) and mtDNA COI gene sequences (Wang et al. 2020). Although previous studies (Gao et al. 2023; Ma et al. 2012, 2011) provided valuable information in resource management and breeding of mud crabs, there are still some shortcomings. For example, the limited molecular markers in most previous studies have had difficulty reflecting the genetic diversity at the whole genome‐wide level and identifying candidate genes related to natural selection and adaptive evolution. Moreover, it could not detect the male‐mediated gene flow based solely on maternally inherited mitochondrial markers, such as mtDNA (Luo et al. 2013; Osellame et al. 2012).

With the rapid development of high‐throughput sequencing and genotyping technologies, whole‐genome resequencing has been widely employed to investigate genetic diversity, population divergence, and candidate genes of economically important traits (Harish et al. 2024; Kurland et al. 2024; Yi et al. 2024). Compared with the limited molecular markers, whole‐genome resequencing data include whole genomic variants, even causal mutations, which would contribute to detecting the genomic regions under selection and adaptation. With the greatly decreasing cost of whole‐genome resequencing, genetic diversity analysis based on this approach has been reported in various aquatic species. In Nile tilapia, low and similar levels of genetic diversity were revealed in three farmed populations based on whole‐genome resequencing data, and several growth‐related genes (*NCAPG*, *KLF3*, and *TBC1D1*) in genomic regions under selection were identified, which may serve as potential genomic landmarks linked to traits of biological and commercial significance in Nile tilapia aquaculture (Cádiz et al. 2020). In grass carp, significant differences in genetic structure and population differentiation, and hundreds of genes associated with growth, reproduction, and environmental adaptation were identified between a mono‐female population and two wild populations using whole‐genome resequencing data, which will be helpful for the molecular marker‐assisted breeding and development conservation strategies of grass carp germplasm resources (Zhang et al. 2023). In Pacific white shrimp, 206 genes (*B3GT5*, *CDC42*, and *PXDN*, etc.) in genomic regions under selection were detected in more than one population among two artificially selective and four market‐leading companies breeds using whole‐genome resequencing data, which could be responsible for the improved economic traits and adaptation to modern aquaculture, shedding light on the genetic effects and genomic signatures of selective breeding (Wang et al. 2022). In general, the whole‐genome resequencing data promote the research of genetic diversity, population divergence, and candidate genes of economically important traits.

Runs of homozygosity (ROHs) are continuous homozygous segments that can reveal the demographic evolution of a population over time (Peripolli et al. 2017). Furthermore, ROH also is a common and powerful estimator of whole‐genome inbreeding level to detect recent and ancient inbreeding, because parents that share a relatively recent common ancestor transmit identical haplotypes to their offspring (Dixit et al. 2020; Kirin et al. 2010). Previous studies found that ROH‐based inbreeding was highly correlated with pedigree‐based inbreeding (Biscarini et al. 2020). Currently, ROHs analysis is widely performed in aquatic species to assess the population history and identify candidate genes related to natural selection and adaptive evolution. In turbot, a resistance‐related quantitative trait loci (QTL) for *P. dicentrarichii* (Sma‐USC38) and a growth‐related QTL marker (Sma‐USC223) in ROH islands were identified in wild and domestic populations, providing useful information to estimate genomic inbreeding and identify selective sweeps in turbot (Aramburu et al. 2020). In rainbow trout, both the moderate to high inbreeding coefficient and a QTL associated with bacterial cold disease resistance were revealed in the six French rainbow trout lines based on ROHs, which reflected the degree of inbreeding to guide the genetic breeding programs and provided valuable insights for precision breeding (D'ambrosio et al. 2019). In general, the distribution of ROH patterns across the genome can inform on genomic regions that have potentially been subjected to selective pressure.

To investigate regional population structure and local adaptation of mud crab, a total of 146 samples from four geographical locations in the southeast coastal regions of China were collected for WGS. Based on the genome‐wide SNPs generated from the whole‐genome resequencing data, the population genetic diversity, population structure, and ROHs of mud crabs were detected to understand the genetic characteristics of mud crabs and identify candidate genes associated with local adaptation in mud crabs. This study will provide valuable information for germplasm resource assessment and genome‐assisted breeding.

## 2 Materials and Methods

### 2.1 Data

**FIGURE 1:**
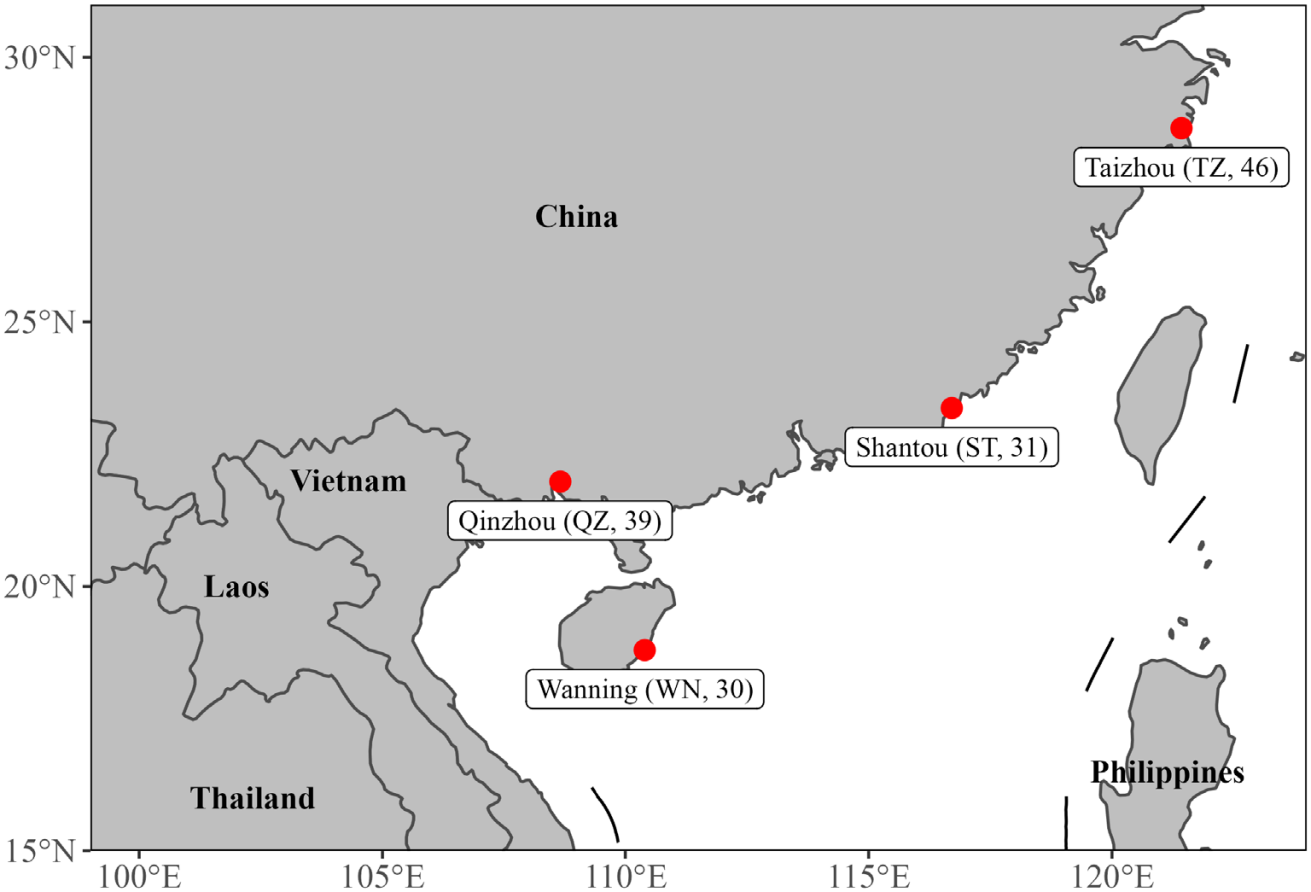
The geographic locations of four mud crab populations in the southeast coastal regions of China. The red dots indicate the sampling locations of mud crab populations. The letters and numbers in parentheses represent the abbreviations for the locations and the number of samples at this location, respectively.

The whole genome resequencing data of 146 mud crabs from four locations distributed in the southeast coastal regions of China were used in this study (Figure 1). There are 30 individuals from Wanning in Hainan, 31 individuals from Shantou in Guangdong, 46 individuals from Taizhou in Zhejiang, and 39 individuals from Qinzhou in Guangxi. In addition, due to the different growth stages of each sample, the phenotypic data was not used for further analysis. DNA from 146 mud crabs was extracted from the muscle tissues using the TIANamp Marine Animals DNA Kit (TIANGEN Biotech Beijing Co. Ltd.). The DNA libraries were prepared and sequenced at BGI‐Shenzhen (Shenzhen 518083, China). Reads were aligned to the *S. paramamosain* reference genome (GCA_035594125.1) using BWA (v.0.7.17). The discovery of variants was made using GATK (v 4.2.0). Detailed information on variant discovery is fully described in our previous study (Ye et al. 2025). After SNP calling and filtering, a total of 146 crabs with 16,714,943 bi‐allelic SNPs were identified. To obtain high‐quality genotype data for further analysis, these SNPs were further filtered using PLINK software (v 1.9) with the following criteria: minor allele frequency (MAF) ≥ 0.05, genotyping call rate ≥ 90%, individual call rate ≥ 90%, and the *p* value of Hardy–Weinberg equilibrium test ≥ 1 × 10^−6^. Finally, a total of 146 crabs with 6,588,886 bi‐allelic SNPs were used for further analysis.

### 2.2 Genetic Diversity Analysis

To assess the genetic diversity of mud crabs from different geographic populations, four characteristic parameters were calculated, including the MAF values, observed heterozygosity (H_O_), expected heterozygosity (H_E_), and genome‐wide nucleotide diversity (π). The MAF, H_O_, and H_E_ were estimated in different geographic populations by PLINK with options “‐freq” and “‐‐hardy”. The π values were calculated by VCFtool (v 0.1.16) with a window size of 20 kb and a sliding step of 10 kb.

### 2.3 Population Structure Analysis

Principal component analysis (PCA), population structure analysis, phylogenetic tree analysis, linkage disequilibrium (LD) analysis, and demographic history analyses were used to investigate the population structure among different populations. In PCA analysis, the principal components (PCs) were estimated by PLINK software with parameter (‐‐pca 5), and the top three PCs were visualized by the R package scatterplot3d. In population structure analysis, SNPs in high linkage disequilibrium were pruned firstly by PLINK with parameter (‐‐indep‐pairwise 50 5 0.5). After pruning, the ancestral population stratification among 146 mud crabs was inferred using Admixture software (Alexander et al. 2009) with remaining SNPs. The optimal ancestral population structure was estimated using ancestral population sizes K (1–5) and choosing the population with the smallest cross‐validation error. In phylogenetic tree analysis, an unrooted neighbor‐joining (NJ) phylogenetic tree was constructed with iTOL software (Letunic and Bork 2024) based on the genetic distance matrix among 146 mud crabs. The pairwise genetic distance matrix between all individuals was estimated as one minus the identity‐by‐state (IBS) value computed by PLINK. Linkage disequilibrium decay was computed by PopLDdecay software (Zhang et al. 2018) with the parameter (‐MaxDist 300) on different populations. The LD decay curve was fitted using the average LD degree over the genetic distance with the R package ggplot2. To investigate population demography of mud crab population, the effective population size of mud crab was estimated by MSMC2 (v 2.1.4) with default parameters (Schiffels and Wang 2020). In addition, we scaled the MSMC estimates using a generation time of 1 year and a mutation rate of 9.2 × 10^−10^, as estimated by OrthoFinder (v 2.5.5) (Emms and Kelly 2019).

### 2.4 Detection of Runs of Homozygosity

Using the WGS data, the ROHs among 146 mud crabs were identified by the sliding window method implemented in the R package detectRUNS with the following parameters: (i) a sliding window of 50 SNPs across the genome, (ii) the proportion of homozygous overlapping windows was 0.05, (iii) the maximum number of heterozygous SNPs in the sliding window was 1, (vi) the maximum number of missing SNPs in the sliding window was 1, (v) the minimum length of an ROH was set to 50 kb, (vi) the required minimum density was set to 1 SNP/50 kb, (vii) the maximum distance between consecutive homozygous SNPs was 100 kb, (viii) the minimum number of SNPs in an ROH was set to be 81, which was calculated according to the formula proposed by Lencz et al. (2007). After ROH detection, the ROHs were categorized into six bins based on their lengths, including 0–0.1, 0.1–0.2, 0.2–0.4, 0.4–0.8, 0.8–1.6, and > 1.6 Mb. In addition, the number and length of ROHs per individual, the percentage of ROH segments per chromosome, and the density distribution were analyzed to reveal the characterization of ROHs in 146 mud crabs.

### 2.5 Estimation of Genomic Inbreeding Coefficient

We computed genomic inbreeding coefficients based on ROHs (*F*_ROH_) and homozygous sites (*F*_HOM_), respectively. *F*_ROH_ was calculated as the total length of ROHs of an individual divided by the total length of the autosomal genome covered by SNPs, as follows:

$$F_{\mathrm{ROH}}=\sum L_{\mathrm{ROH}}/L_{\text{genome}}$$

$\sum L_{\mathrm{ROH}}$ is the sum length of all ROHs with segments longer than 50 kb; and $L_{\text{genome}}$ is the sum length of all chromosomes, because of the lack of determined sex chromosome in mud crab (Cui, Guan, et al. 2021; Shi et al. 2018; Waiho et al. 2019; Zhang et al. 2024).

*F*_HOM_ was estimated by PLINK with option “‐‐het” based on the following formula:

$$F_{\mathrm{HOM}}=\left(C_{\text{observed}}-C_{\text{expected}}\right)/\left(N-C_{\text{expected}}\right)$$

$C_{\text{observed}}$ and $C_{\text{expected}}$ are observed and expected homozygous genotype counts of each sample, respectively; and N is the total number of samples.

### 2.6 Identification of Potential ROH Islands and Candidate Genes

To identify the potential ROH islands, the percentage of SNP occurrences in all ROHs was calculated and plotted against the position of SNPs along 49 chromosomes. According to Xu et al. (2019), SNPs with a percentage higher than 50% of occurrence were defined as high‐frequency SNPs and a hint of a potential ROH island. To evaluate the differences in selection pressure among ROH islands, ROHs, and non‐ROH regions, we employed multiple approaches to assess selection pressure, including pooled heterozygosity (Hp), Tajima's *D*, and π. The Tajima's D and π values were calculated using VCFtools (v 0.1.16) with a 40 kb window size, but the calculation of π values needs a 20 kb sliding step as an additional parameter. The Hp values were calculated by following the formula in a sliding 40 kb window with a 20 kb sliding step:

$$\mathrm{Hp}=2\sum n_{\mathrm{MAJ}}\sum n_{\mathrm{MIN}}/{\left(\sum n_{\mathrm{MAJ}}+\sum n_{\mathrm{MIN}}\right)}^{2}$$

$n_{\mathrm{MAJ}}$ and $n_{\mathrm{MIN}}$ are the numbers of reads corresponding to the most and least frequently observed allele of each SNP (Rubin et al. 2010). Finally, the high‐frequency SNPs were annotated to identify candidate genes in ROH islands using SnpEff software (Cingolani et al. 2012). To further obtain functional annotation information of candidate genes, the protein sequences of candidate genes were queried using the eggNOG‐mapper software (v 2.1.12) (Huerta‐Cepas et al. 2017) with eukaryote database (v 5.0.2).

## 3 Results

### 3.1 The Genetic Diversity Analysis of Mud Crabs Among Different Geographic Populations

The MAF, Ho, H_E_, and π values of QZ, ST, TZ, and WN populations were shown in Table 1. Results showed that the number of SNPs with MAF ≥ 0.05 was similar in QZ, TZ, and WN populations, ranging from 6,067,714 to 6,069,429 with the same proportion (0.921), which was slightly higher than that in the ST population (5,706,649 with the proportion of 0.866). The number of SNPs with MAF ≥ 0.2 was 2,264,707 in the QZ population and 2,269,400 in the ST population, both with a proportion of 0.344, which was slightly lower than that in TZ and WN populations (2,277,048 with the proportion of 0.346). The π values were 0.0016 in TZ and WN populations, 0.00158 in the ST population, 0.00157 in the QZ population, and 0.00159 in all samples. The values of H_O_ ranged from 0.248 to 0.257 in these populations, approximately equal to the values of H_E_ (0.260–0.265). Overall, the mud crab populations from different geographic regions in the southeast coast of China have a similar level of genetic diversity and are approximately in Hardy–Weinberg equilibrium, but the level of polymorphism was low.

**TABLE 1:** The summary of genetic diversity of mud crabs in different geographic populations.

POP	*N*	NSNP_0.05_	NSNP_0.2_	PN_0.05_	PN_0.2_	H_O_	H_E_	π
QZ	39	6,067,714	2,264,707	0.921	0.344	0.248	0.260	0.00157
ST	31	5,706,649	2,269,400	0.866	0.344	0.252	0.260	0.00158
TZ	46	6,069,429	2,277,048	0.921	0.346	0.257	0.263	0.00160
WN	30	6,069,429	2,277,048	0.921	0.346	0.257	0.263	0.00160
ALL	146	6,588,886	2,252,177	1.000	0.342	0.254	0.265	0.00159

Abbreviations: H_E_, expected heterozygosity; H_O_, observed heterozygosity; *N*, the number of samples; NSNP_0.05/0.2_, the number of SNPs with MAF ≥ 0.05 or 0.2; PN_0.05/0.2_, the ratio of SNPs with MAF ≥ 0.05 or 0.2; POP, population; π, nucleotide diversity.

### 3.2 Population Structure Analysis

**FIGURE 2:**
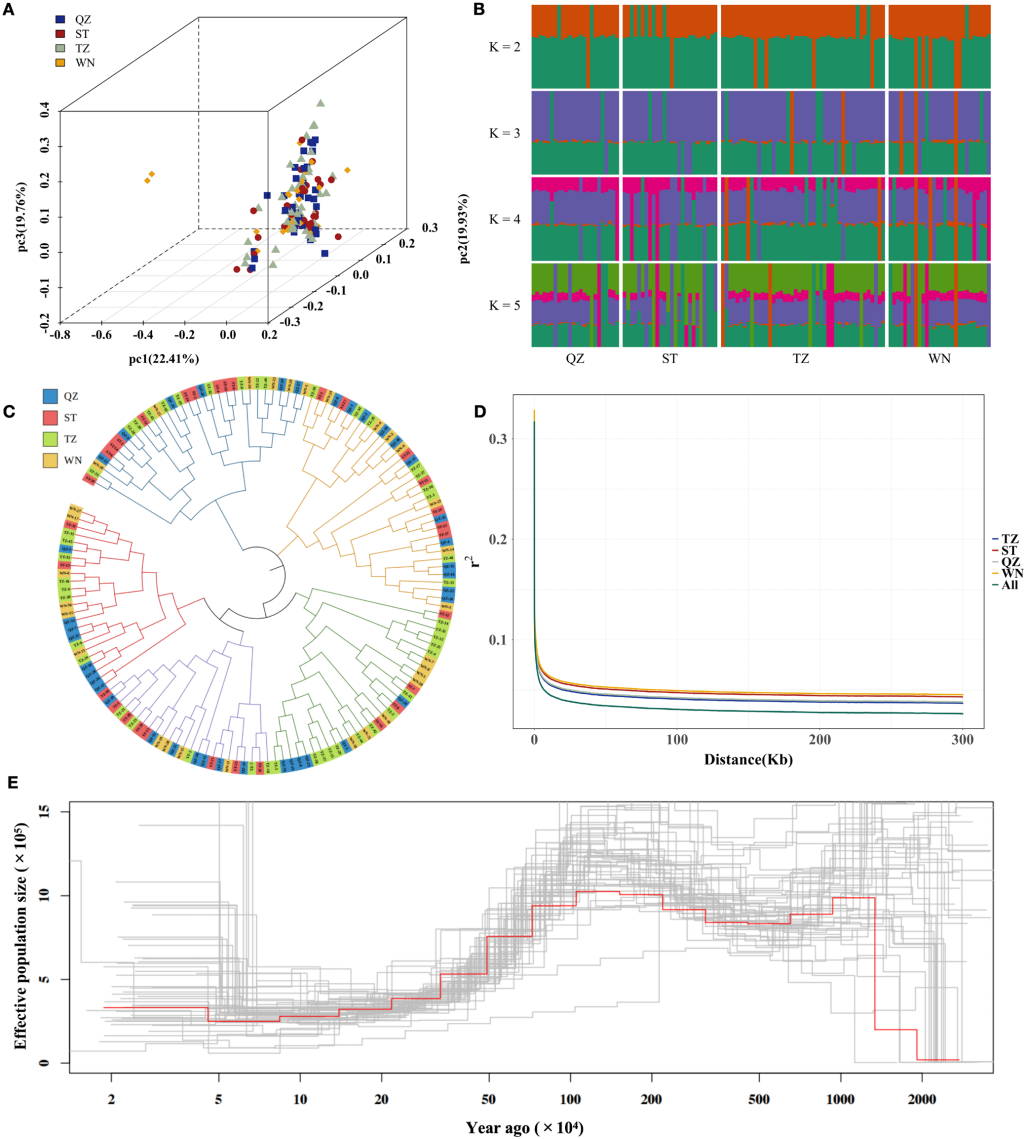
The population genetic structure analysis of 146 mud crabs from four geographic populations (QZ, ST, TZ, and WN) in the southeast coastal regions of China. (A) Three‐dimensional scatterplots of 146 mud crabs based on principal components 1–3 (pc1‐pc3). (B) Genetic structure analysis of 146 mud crabs with changing ancestral populations from *K* = 2 to *K* = 5. (C) Phylogeny tree of 146 mud crabs using the neighbor‐joining method. (D) Decay of linkage disequilibrium (LD) with physical distance in different mud crab populations. (E) Changes in effective population sizes through time.

To understand the population genetic structure of the mud crab populations in the southeast coastal regions of China, the PCA, population structure, phylogenetic tree, LD analysis, and demographic history analyses were performed in this study (Figure 2). The PCA result showed that nearly all individuals were clustered into a single group based on the first three principal components, except for two individuals from the WN population. The contributions of the first three principal components (pc) were 22.41%, 19.93%, and 19.76% respectively, accumulating 62.10% contributions (Figure 2A). Furthermore, the population admixture analysis with *K* values ranging from 1 to 5 revealed that most individuals across four populations exhibited similar ancestral components, without significant population stratification among different geographical populations (Figure 2B). The error values of cross‐validation of admixture analysis with different K also showed that the optimal number of ancestral populations was one (Table S1). Although the phylogenetic tree clustered all individuals into five branches (Figure 2C), the individuals from different geographic regions were not separated into distinct branches. Instead, all individuals were dispersed across various branches, which indicated genetic homogeneity across these populations, consistent with the findings from the PCA and admixture analyses. According to the admixture analyses and phylogenetic tree, two distinct individuals of the WN population were not significantly differentiated from the rest of the individuals. The result of LD analysis showed that LD declined rapidly with increasing physical distance across all populations (Figure 2D). As the distance between variants increased, LD between SNPs declined fastest in all samples, followed by TZ and QZ, and the slowest in ST and WN. Demographic history analyses revealed a sharp decrease in the effective population size (*N*_e_) around 1 million years ago, reflecting a historical bottleneck, followed by a population expansion starting around 50,000 years ago (Figure 2E). In general, the different geographic mud crab populations in the southeast coastal regions of China did not exhibit significant genetic differentiation.

### 3.3 Characteristic of Runs of Homozygosity in Mud Crabs

**FIGURE 3:**
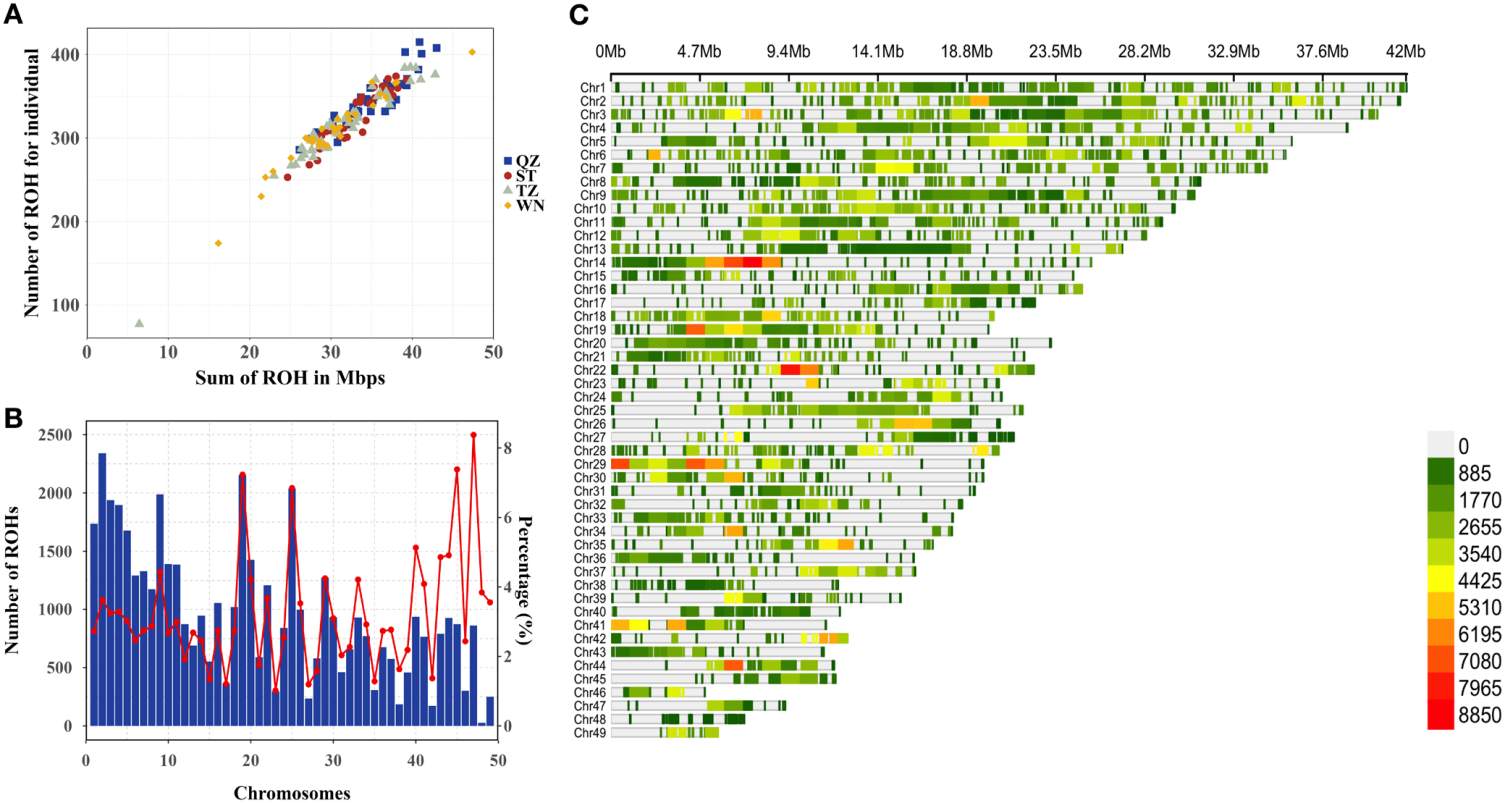
Characteristic of runs of homozygosity (ROHs) in mud crabs. (A) The relationship between total genomic length (Megabases) covered by ROH per individual (*X* axis) and the total number of ROH per individual (*Y* axis). (B) The number and percentage (red line) of ROHs across the 49 chromosomes. (C) ROHs density distribution of 49 chromosomes in mud crab (*S. paramamosain*) within 1 Mb window size.

To understand the characteristics of ROHs in mud crabs from southeast coastal regions of China, the genome‐wide run of homozygosity analysis among 146 mud crabs was performed using WGS data. Results showed that a total of 47,142 ROHs with an average length of 0.1 Mb (ranging from 0.050 to 1.08 Mb) were identified (Table S2). Most of the ROHs (31,196 with the proportion of 66.17%) were shorter than 0.1 Mb, followed by ROHs with the range of 0.1–0.4 Mb (15,753 with the proportion of 33.42%), and a small portion of ROHs (193 with the proportion of 0.41%) exceeded 0.4 Mb in length (Table S3), suggesting a low degree of inbreeding within the recent population. A clear positive correlation was revealed between the total number of ROHs and the cumulative length of ROHs (Figure 3A). Notably, most individuals (98.63%) fell within a concentrated cluster, possessing 200–400 ROHs with a total length of 20–40 Mb, suggesting that individuals exhibited similar levels of homozygosity in terms of both the number and length of ROHs. The number, percentage, and density of ROHs across the 49 chromosomes showed that the ROHs distribution among the 146 individuals varied notably by chromosome (Figure 3B,C). For example, chromosome 2 had the highest number of ROHs (2341), but the total ROH length was only 3.62% of the whole chromosome length. Conversely, chromosome 47, despite being the shortest chromosome, showed a high percentage of total ROH length at 8.37% with a total of 862 ROHs. Additionally, chromosome 48 had the fewest ROHs (26) with an ROH length percentage of 3.84%. In general, the number and length of ROHs were similar in 146 crabs, but unevenly distributed across 49 chromosomes.

### 3.4 Evaluation of Genomic Inbreeding Coefficients Based on ROHs and Homozygous Sites

**FIGURE 4:**
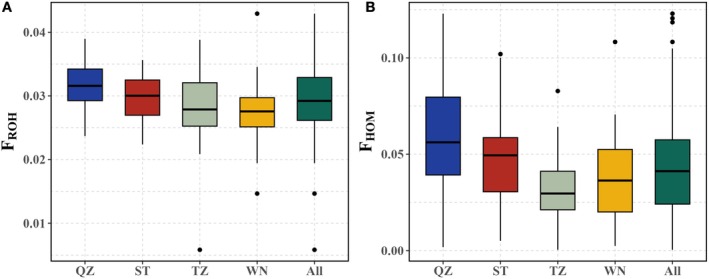
The genomic inbreeding coefficients of mud crabs in different geographic populations. (A) Inbreeding coefficient based on ROHs (*F*_ROH_). (B) Inbreeding coefficient based on homozygous sites (*F*_HOM_).

To assess the inbreeding degree of mud crabs in the southeast coastal regions of China, we evaluated the genomic inbreeding coefficients of mud crabs in different geographic populations (QZ, ST, TZ, and WN) based on ROHs (*F*_ROH_) and homozygous sites (*F*_HOM_) using WGS data. The average values of *F*_ROH_ for QZ, ST, TZ, and WN populations were 0.0315, 0.0298, 0.0284, and 0.0275, respectively, while the average values of *F*_HOM_ were 0.0589, 0.0458, 0.0267, and 0.0243, respectively (Figure 4). Comparing with the average inbreeding rate (0.0293) evaluated by *F*_ROH_, the average inbreeding rate (0.0389) of *F*_HOM_ was slightly higher (*p* < 0.001). However, the trend of *F*_ROH_ and *F*_HOM_ in different geographic populations was similar, showing that the QZ population had the highest value of inbreeding coefficient, while the WN population presented the lowest value. In general, the inbreeding of mud crab populations from different geographic regions on the southeast coast of China was very low.

### 3.5 Identification of Potential ROH Islands and Related Candidate Genes

**FIGURE 5:**
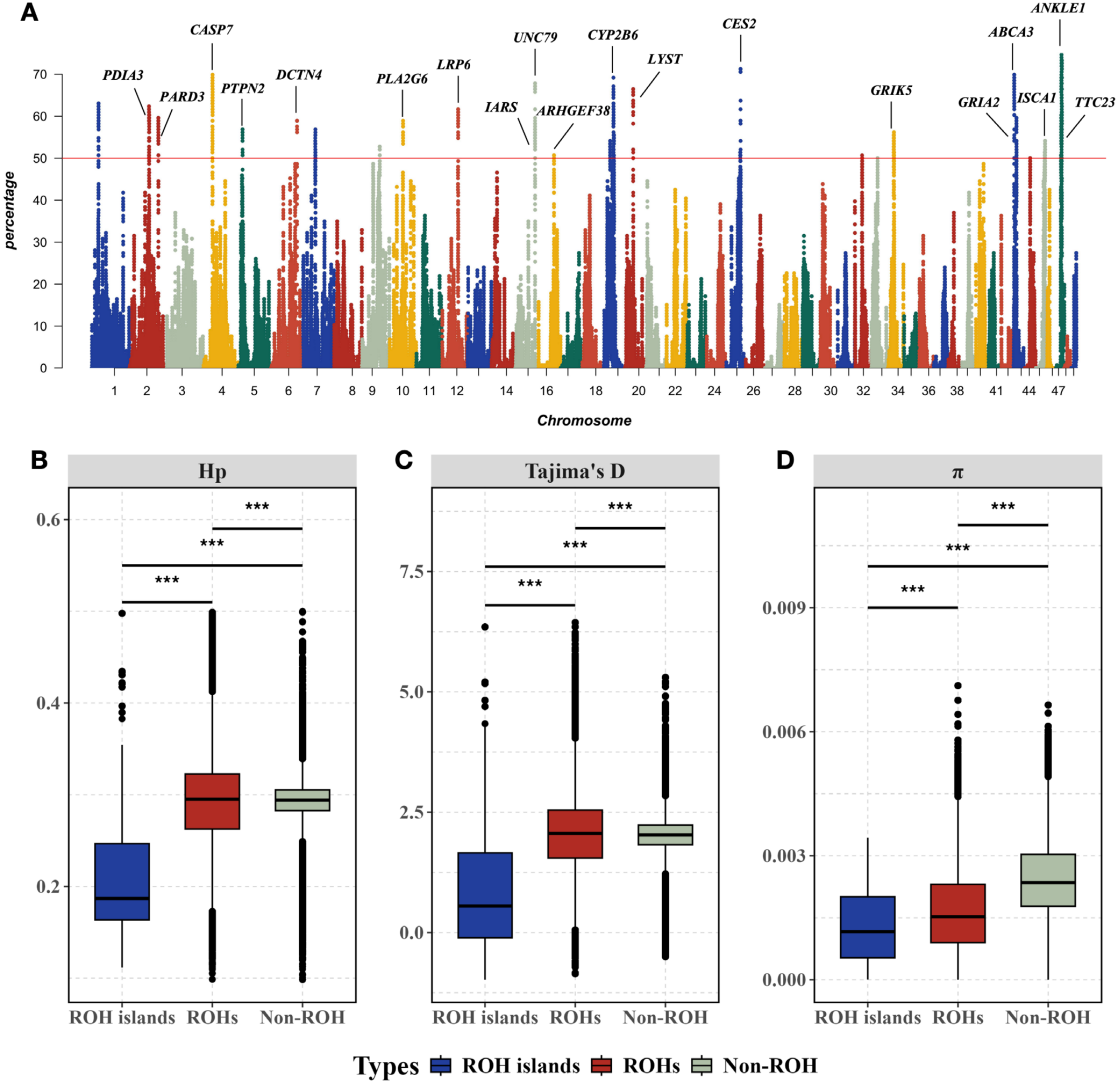
Distribution of high‐frequency SNPs and selective pressure comparison across genome in mud crabs. (A) Manhattan plot of the frequency of SNPs within runs of homozygosity (ROHs) region among 146 mud crabs. The red line represents the thresholds (50%) to identify high‐frequency SNPs of ROH islands. (B) The Hp values of ROH islands, ROHs and non‐ROH regions. (C) The Tajima's *D* values of ROH islands, ROHs and non‐ROH regions. (D) The π values of ROH islands, ROHs and non‐ROH regions. *** represents *p* < 0.001.

To identify the potential ROH islands and related candidate genes, the percentage of SNP occurrences within all ROHs was calculated (Figure 5A). Results showed that a total of 10,246 SNPs with an occurrence rate of greater than 50% in the ROHs were located on 19 chromosomes, such as chromosomes 1, 2, 4, and so on. Among them, chromosome 47 had the most high‐frequency SNPs (2498), while chromosome 32 only presented 1 high‐frequency SNP. Compared with ROHs and non‐ROH regions, SNPs within ROH island regions exhibited significantly lower Hp, Tajima's *D*, and π values (*p* < 0.001) (Figure 5B–D), showing reduced genetic diversity and heterozygosity, which suggested ROH islands were under stronger selective pressure than ROHs and non‐ROH genomic regions. After gene mapping and functional annotation, a total of 29 candidate genes in ROH islands were identified, and the detailed information was summarized in Table 2. Among these genes, two genes related to growth and development were located on chromosome 15, namely the isoleucinyl‐tRNA synthetase (*IARS*) gene and the sodium leak channel NALCN accessory subunit uncoordinated 79 (*UNC79*) gene. Besides, six genes (*PARD3*, *CASP7*, *PTPN2*, *FMNL2*, *HMGB2*, and *MLH3*) regulated a variety of cellular processes, including cell growth, differentiation, cytokinesis, and cell death. In addition, the carboxylesterase 2 (*CES2*) gene and cytochrome P450 Family 2 subfamily B member 6 (*CYP2B6*) gene were involved in drug metabolism, participating in cholesterol ester metabolism and synthesis of cholesterol. Taken together, these genes may play an important role in the adaptive evolution of mud crabs, especially in growth.

**TABLE 2:** Characterization and function annotations of 29 candidate genes.

Gene name	Position	Number of SNPs in ROHs	Description
*PDIA3*	2:32264414–32,276,473	36	Protein disulfide isomerase family A member 3
*PARD3*	2:32289797–32,384,220	20	Par‐3 family cell polarity regulator
*CASP7*	4:10248324–10,275,627	35	Cysteine‐aspartic acid protease (caspase) 7
*HM13*	4:10275666–10,308,170	99	Histocompatibility minor 13
*TIMM9*	4:10308493–10,313,085	45	Translocase of inner mitochondrial membrane 9
*NFXL1*	4:10316259–10,334,946	78	Nuclear transcription factor, X‐box binding like 1
*PTPN2*	5:4684610–4,839,316	87	Protein tyrosine phosphatase non‐receptor type 2
*FMNL2*	6:29049266–29,067,057	46	Formin like 2
*DCTN4*	6:29073263–29,086,261	36	Dynactin subunit 4
*HOGA1*	6:29090657–29,108,626	11	4‐hydroxy‐2‐oxoglutarate aldolase 1
*HMGB2*	10:15137532–15,142,142	14	High mobility group box 2
*SAP30L*	10:15145394–15,147,982	18	SAP30 like
*PLA2G6*	10:15173360–15,222,576	53	Phospholipase A2 group VI
*LRP6*	12:17717024–17,800,290	94	LDL receptor related protein 6
*UNC79*	15:21929690–22,014,375	227	Unc‐79 homolog, NALCN channel complex subunit
*IARS*	15:22017320–22,041,066	6	Isoleucyl‐tRNA synthetase
*ARHGEF38*	16:17426260–17,549,742	111	Rho guanine nucleotide exchange factor 38
*CYP2B6*	19:10803443–11,196,028	974	Cytochrome P450 Family 2 subfamily B member 6
*SF3B2*	20:9184310–9,188,080	20	Splicing factor 3b subunit 2
*MLH3*	20:9191015–9,198,634	62	MutL homolog 3
*NTHL1*	20:9199913–9,213,546	36	Nth like DNA glycosylase 1
*LYST*	20:9214374–9,224,447	20	Lysosomal trafficking regulator
*CES2*	25:15951990–16,001,221	29	Carboxylesterase 2
*GRIK5*	34:6511119–6,533,267	333	Glutamate ionotropic receptor kainate type subunit 5
*ABCA3*	43:3165117–3,281,014	282	ATP binding cassette subfamily A member 3
*GRIA2*	43:3300632–3,359,676	421	Glutamate ionotropic receptor AMPA type subunit 2
*ISCA1*	45:9701078–9,729,475	27	Iron–sulfur cluster assembly 1
*TTC23*	47:6115114–6,265,659	266	Tetratricopeptide repeat domain 23
*ANKLE1*	47:6482621–6,499,270	25	Ankyrin repeat and LEM domain containing 1

## 4 Discussion

Understanding the genetic diversity, population structure, and population history of mud crabs would provide valuable insights for conserving, managing, and improving genetic resources. In this study, a total of 146 mud crabs from four geographic populations were used to perform whole genome resequencing to explore the genetic diversity, population genetic structure, and patterns of ROHs of mud crabs in the southeast coastal regions of China. To our knowledge, this is the first time using WGS data to investigate the genetic diversity, population divergence, and candidate genes related to adaptive evolution in mud crabs.

### 4.1 The Genetic Diversity and Population Structure of Mud Crab

The genetic diversity and population structure analysis with WGS data revealed a low level of polymorphism and genetic differentiation among mud crabs from southeast coastal areas of China. In this study, the values of π (0.00159), H_O_ (0.254), and H_E_ (0.265) in all samples (Table 1) indicated the low level of polymorphism within the mud crab population in the southeast coastal areas of China. These results are similar to previous studies on mud crab (Ma et al. 2011; Wang et al. 2020), swimming crab (Duan et al. 2022), and European green crab (Tepolt et al. 2022), but relatively lower than Chinese mitten crab (Su et al. 2023), orange mud crab (Habib et al. 2022) and giant mud crab (Rumisha et al. 2017). It might result from overexploitation or historical bottlenecks driven by increased market demand (Grant and Bowen 1998; Rumisha et al. 2017). Nevertheless, the similar values of H_O_ (0.254) and H_E_ (0.265) suggested that the population was in approximate Hardy–Weinberg equilibrium and had not experienced strong artificial or natural selection (Abramovs et al. 2020). Additionally, it is evident that commercially exploited marine species exhibited low levels of genetic differentiation largely due to their high dispersal capacities and the lack of physical barriers in the marine environment (Gandra et al. 2021). Moreover, the *F*_ST_ values among four populations were generally low, further confirming that there was no significant genetic differentiation among four populations (Figure S1). This pattern has been documented in several species, including horseshoe crab (Pedrosa‐Gerasmio et al. 2025), mitten crab (Su et al. 2023), and oysters (Calla et al. 2025). According to the results of the PCA, population structure, phylogenetic tree, and LD analysis, mud crabs from different geographic populations were intermixed without obvious geographical differentiation, indicating the low genetic differentiation among mud crabs from southeast coastal areas of China. These results also are similar to previous studies in mud crab (Ma et al. 2011; Wang et al. 2020). The observed weak genetic differentiation could be attributed to the high dispersal capability of mud crab and minimal barriers in a marine environment. Theoretically, species with greater dispersal potential tend to exhibit lower levels of interpopulation genetic structure. In mud crab, frequent migrations among ocean basins and coastal regions likely promote gene flow and genetic exchange (Beheregaray and Sunnucks 2001; Ma et al. 2012; Wang et al. 2020). Furthermore, as an important economic aquatic species with high market demand, mud crab is frequently transported across regions, which might further reduce the genetic differentiation of mud crabs across geographical locations (Su et al. 2023). Moreover, selective fishing may also contribute to the observed weak genetic differentiation (Gandra et al. 2021). Commercial fishing often targets individuals with specific traits (e.g., large body size), which may act uniformly across regions, thereby homogenizing genetic structure and reducing detectable geographic differentiation (Alós et al. 2016). In addition, ancient bottleneck indicated by N_e_ decline may have substantially reduced genetic diversity by intensifying genetic drift due to a reduced population size, with its legacy still detectable in present low polymorphism levels (Lucena‐Perez et al. 2021). Overall, the mud crab populations have a low level of polymorphism and weak genetic differentiation but still maintain sufficient genetic variation along the southeast coastal areas of China.

### 4.2 The Runs of Homozygosity of Mud Crab

ROHs are continuous homozygous segments that can reveal the long‐term demographic evolution (Peripolli et al. 2017) and whole‐genome inbreeding level for a population (Dixit et al. 2020; Kirin et al. 2010). In this study, a total of 47,142 ROHs were identified in mud crabs from the southeast coastal areas of China, with 66.17% of ROHs being shorter than 0.1 Mb in length (Table S2). Compared with Pacific White Shrimp (Wang et al. 2022), Nile tilapia (Robledo et al. 2024), and Coho Salmon (Yoshida et al. 2020), the ROHs have shorter segments and a much smaller number in mud crabs, which might result from the different criteria used for ROHs detection and the population history. When setting the minimum length of ROH from 25 kb to 100 kb, the proportion of shorter ROHs decreased continuously (Table S3). In addition, the length and number of ROHs provide important insights into the history of inbreeding events, with long ROHs typically indicating close consanguinity in recent generations, and short ROHs arising from recombination events across generations and tracing back to more remote ancestors (Aramburu et al. 2020; Purfield et al. 2012). Therefore, the high proportion of short ROHs (66.17%) observed in mud crab populations suggested the occurrence of distant ancestral inbreeding events and infrequent inbreeding events in recent generations, further evidenced by a significant reduction in N_e_ around 1 million years ago, followed by a subsequent recovery. Furthermore, the low inbreeding coefficients derived from ROHs (*F*_ROH_) of mud crab populations were similar to Chinese mitten crab (Wang et al. 2025) and Atlantic blue crab (Cushman and Darden 2017). Notably, the low inbreeding coefficients derived from ROHs (*F*_ROH_), consistent with the predominance of short ROHs, suggested that the mud crab population had enough effective population size, similar to the findings from the previous study (Zhao et al. 2021). Moreover, the low levels of inbreeding observed in mud crabs could indicate that wild broodstock continue to be widely utilized in mud crab aquaculture. Additionally, similar patterns were exhibited in terms of the total number and length of ROHs in mud crabs from different geographic populations that also suggested weak genetic differentiation among mud crabs from southeast coastal areas of China.

### 4.3 The Candidate Genes for Adaptive Evolution in Mud Crab

Positive selection drives the fixation of advantageous alleles, leading to extended homozygosity around selected loci, for which genomic regions under strong selection pressure tend to generate ROH islands, showing reduced genetic diversity and elevated homozygosity (Pemberton et al. 2012; Purfield et al. 2017). For example, substantial differences in the distribution of ROH islands have been observed between domestic and wild turbot populations, with several QTLs associated with ROH islands linked to growth and disease resistance traits (Aramburu et al. 2020). In large yellow croaker, the extent of haplotype homozygosity in the positively selected regions increased after two successive rounds of genomic selection against the parasite *Cryptocaryon irritans*, during which key candidate genes significantly associated with the resistance to *C. irritans* were identified (Zhao et al. 2025). Accordingly, ROH islands could serve as indicatives of adaptive responses to environmental or selective pressures, helping to pinpoint candidate genomic regions under selection and search for candidate genes related to important traits (Rodríguez‐Ramilo et al. 2021). Notably, it is critical to select an appropriate threshold to identify ROH islands. However, no consensus threshold for defining ROH islands has been established in previous studies (Sarviaho et al. 2024). The most common approach is to classify the top 1% of SNPs with the highest occurrence frequency as potential ROH islands, with reported frequency thresholds of SNPs in ROHs ranging from 10% (Mastrangelo et al. 2018) to 67.8% (Li et al. 2024). In this study, based on a threshold of 50%, a total of 29 candidate genes in potential ROH islands of mud crabs were identified, particularly the *IARS* and *UNC79*, which have been reported associated with growth and development in other species. The *IARS* gene encodes an enzyme essential for catalyzing the binding of isoleucine to its corresponding tRNA during protein synthesis (Jiang et al. 2024). The mutations of the *IARS* gene cause growth retardation, intellectual disability, and liver disorders in humans (Kopajtich et al. 2016; Orenstein et al. 2017), and similar defects in livestock, such as hereditary weak calf syndrome in Japanese Black cattle (Ikeda et al. 2017). The *UNC79* gene encodes a component of the hetero‐tetrameric NALCN sodium leak channel, which regulates resting membrane potential and mediates sodium leak currents. Mutations of NALCN channel components, including UNC79, resulted in neurodevelopmental diseases (Kang and Chen 2022). Specifically, UNC79 affects circadian rhythms, sleep, starvation resistance, and metabolic regulation, with its mutation causing growth retardation and developmental delays (Bayat et al. 2023; Murakami et al. 2021). These findings suggest that *IARS* and *UNC79* may play critical roles in biological processes such as growth and development in mud crabs, potentially contributing to adaptive evolution. However, further research is needed to elucidate the precise functions of these genes in mud crab biology and their possible adaptive significance. In general, the identification of these genes within ROH islands provides valuable molecular markers for future studies on related traits, offering a promising direction for validating the genetic basis in mud crabs.

### 4.4 Practical Application and Potential Implications

Genetic diversity is fundamental for species adaptation, evolutionary processes, and long‐term survival (Fageria and Rajora 2013). Systematic analysis of molecular diversity within genetic resources is crucial for developing effective conservation strategies and breeding programs (Yirgu et al. 2023). Currently, global genetic diversity loss has become an increasing concern across numerous species (Shaw et al. 2025). In this study, although limited genetic differentiation and inbreeding among geographically distinct populations were detected, the overall low polymorphism levels pose a serious challenge for both resource conservation and breeding initiatives, which could further exacerbate if left unaddressed. Therefore, it is imperative to conserve and enhance genetic diversity to ensure the long‐term viability and adaptability of mud crab, because reduced genetic variability limits evolutionary adaptation and response to selection in breeding programs, making the preservation of genetic diversity essential for sustainable resource management (Aramburu et al. 2020). To prevent further loss of genetic diversity and maintain genetic biodiversity, targeted conservation measures should be implemented. One key approach is to implement fishing restrictions, such as establishing closed fishing seasons, to allow mud crab populations to recover and regenerate naturally (Sadler et al. 2023), which would give populations the opportunity to stabilize and potentially increase their genetic diversity. Effective conservation actions, coupled with careful management, will be essential for ensuring the long‐term sustainability of mud crab populations and preserving genetic resources.

Besides, the findings of this study also provide practical insights for the design and implementation of genomic selection (GS) strategies in mud crab. The weak genetic differentiation observed among mud crab populations from different geographic regions supports the feasibility of combining multiple populations to establish a larger and more genetically diverse reference population for GS. Since reference population size is a key factor influencing GS accuracy, combining different populations in GS is an attractive way to improve prediction accuracy (Ye et al. 2022). Furthermore, the weak subpopulation structure in the combined populations may mitigate confounding effects due to stratification, further improving the accuracy of GS (Minamikawa et al. 2018). Therefore, based on the weak genetic differentiation, combining multiple regional populations is a promising strategy for constructing an effective reference population in mud crab breeding programs.

Additionally, the results of this study offer important implications for genetic breeding in mud crab. Estimations of LD extent and decay are critical for optimization of SNP arrays in GS (Barría et al. 2019). Theoretically, QTLs or chromosome segments contributing to phenotypic variation are expected to be in LD with at least one marker locus (Meuwissen et al. 2001). Populations with strong LD require fewer markers to capture genetic variance due to the reduced number of independent segments, offering opportunities to develop cost‐effective and lower‐density SNP arrays without compromising prediction accuracy (Wientjes et al. 2013). Moreover, comparative analyses of LD extent and decay can also help identify genomic regions under different selective pressures and elucidate the genetic basis of economically important traits (López et al. 2015). Importantly, the comparative analysis of selection pressure among ROH islands, ROHs, and non‐ROH regions revealed distinct signatures of selective sweeps in ROH islands regions, suggesting ROH islands may harbor genes under intense artificial or natural selection (Belay et al. 2024; Zhang et al. 2021). Notably, 29 candidate genes were identified in ROH islands, including two genes associated with growth and development. These candidate genes offer promising targets for the development of customized SNP arrays, which have been shown to enhance the power and accuracy of both GWAS and GS (Anilkumar et al. 2023). Genomic regions or markers identified through LD and ROH analyses and associated with key traits, such as growth rate, disease resistance, and reproductive performance, can be further functionally validated and subsequently incorporated into selective breeding programs through marker‐assisted selection (MAS) and GS. Such loci could be utilized as feature markers to enhance the accuracy of GS, as demonstrated in blotched snakehead, where the inclusion of trait‐associated SNPs increased the accuracies of genomic prediction for weight and total length by 35.90% and 26.67%, respectively (Cui et al. 2025). Alternatively, they could function as genetic markers linked to major QTLs that influence traits, as seen in the use of DNA markers linked to QTLs affecting host resistance to infectious pancreatic necrosis virus (IPNV) in Atlantic salmon (Moen et al. 2009). Overall, these findings would enable more efficient and targeted improvement strategies in selective breeding programs for mud crab.

## 5 Conclusions

A low level of polymorphism and genetic differentiation was revealed in mud crabs from southeast coastal areas of China using WGS data. Notably, a total of 29 candidate genes with high‐frequency SNPs in potential ROH islands were identified, including growth and development‐related genes (*IARS* and *UNC79*). In general, these findings will provide valuable insights for conserving, managing, and improving the genetic resources of mud crabs (*S. paramamosain*).

## Ethics Statement

The authors have nothing to report.

## Consent

The authors have nothing to report.

## Conflicts of Interest

The authors declare no conflicts of interest.

## Supporting information


**Figure S1:** *F*_ST_ (Fixation statistic) analysis among four populations.
**Table S1:** CV error corresponding to each K value in Admixture analysis.
**Table S3:** ROH lengths category based on different minimum length (25 kb, 50 kb and 100 kb) of ROH.

**Table S2:** Information of ROHs in 146 mud crabs (Supporting Informations‐Table S2.xlsx).

## Data Availability

The genotype data of 146 mud crabs has been uploaded to the Dryad repository (https://doi.org/10.5061/dryad.3r2280gvq).
