# Supplementary material for: Whole‐Genome Resequencing Provides Novel Insights Into the Genetic Diversity, Population Structure, and Patterns of Runs of Homozygosity in Mud Crab (Scylla paramamosain)
Source: Evol Appl. 2025 Sep 3;18(9):e70153. doi: 10.1111/eva.70153 (PMC12406289; doi:10.1111/eva.70153)
Supplement: Supplementary file 1 — Figure S1: F ST (Fixation statistic) analysis among four populations. Table S1: CV error corresponding to each K value in Admixture analysis. Table S3: ROH lengths category based on different minimum length (25 kb, 50 kb and 100 kb) of ROH. [file EVA-18-e70153-s002.docx]

# Supplementary materials

**
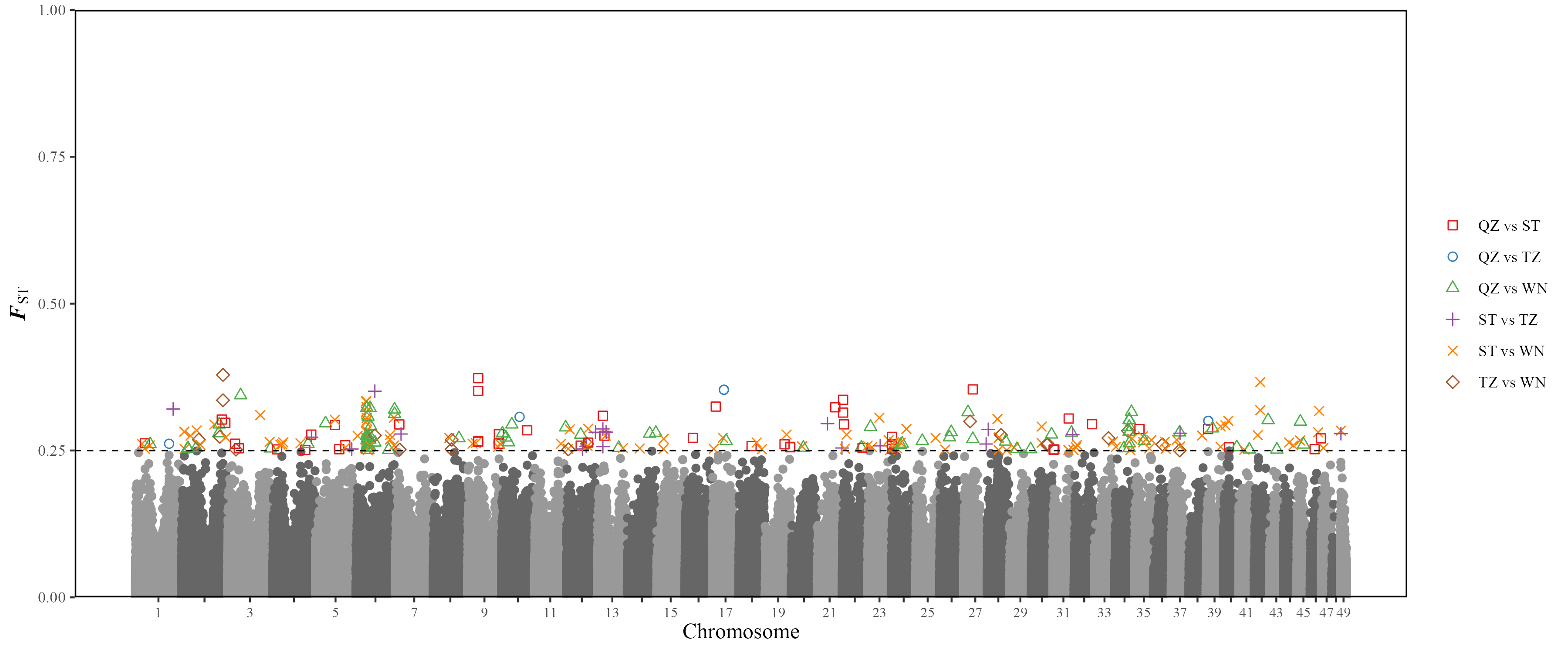
**

**Figure S1.** *F*_ST_ (Fixation statistic) analysis among four populations.

**Table S1.** CV error corresponding to each K value in Admixture analysis.

| **K** | **CV error** |
| --- | --- |
| 1 | 0.52726 |
| 2 | 0.55343 |
| 3 | 0.58604 |
| 4 | 0.61479 |
| 5 | 0.64743 |

**Table S2.** Information of ROHs in 146 mud crabs (Supplementary Materials-Table S2.xlsx).

**Table S3.** ROH lengths category based on different minimum length (25 kb, 50 kb and 100 kb) of ROH.

| **Length of ROHs (Mb)** | **Number of ROHs** | | |
| --- | --- | --- | --- |
|  | **25 kb** | **50 kb** | **100 kb** |
| 0-0.1 | 86304 | 31196 | 0 |
| 0.1-0.2 | 12927 | 12927 | 12927 |
| 0.2-0.4 | 2826 | 2826 | 2826 |
| 0.4-0.8 | 188 | 188 | 188 |
| 0.8-1.6 | 5 | 5 | 5 |
